# Microfluidic-Based Whole-Cell Biosensor Systems—Challenges and Future Applications

**DOI:** 10.3390/bios16030173

**Published:** 2026-03-20

**Authors:** Niklas Fante, Alexander Grünberger

**Affiliations:** 1 Multiscale Bioengineering, Faculty of Technology Bielefeld University, Universitätsstraße 25, 33615 Bielefeld, Germany; niklas.fante@uni-bielefeld.de; 2 Center for Biotechnology (CeBiTec), Bielefeld University, Universitätsstraße 27, 33615 Bielefeld, Germany; 3 Microsystems in Bioprocess Engineering, Institute of Process Engineering in Life Sciences, Karlsruhe Institute of Technology, Fritz-Haber-Weg 2, 76131 Karlsruhe, Germany

**Keywords:** biosensor, biohybrid systems, cell-based biosensor, microbial biosensor, MEMS, µTAS, miniaturization, point-of-care, in situ testing, immobilization

## Abstract

The integration of whole-cell biosensors in miniaturized measuring devices to exploit synergetic effects as small, rapid, cost-effective, sensitive, and highly specific platforms with point-of-care applicability was often discussed in recent years and many different setups have been presented to date. In many cases these setups were envisaged as powerful systems in their respective fields; however, the anticipated success often failed to materialize, and the systems remained a proof-of-concept. We elaborate on the hurdles and possible challenges that have to be overcome for the successful development and application of such systems. Further, we critically discuss and rank the impact of different challenges during system development, application, and commercialization. Finally, we point out possible future applications and conclude future perspectives for whole-cell biosensors integrated into microfluidic platforms.

## 1. Introduction

Sensors are one of the most relevant technical innovations in the control of processes and determination of important environmental conditions. Specialized sensors, known as biosensors, are based on biologically active sensing mechanisms that are triggered by an analyte or environmental condition and coupled to a transducer to convert and generate a measurable response. They show promising potential for the determination and measurement of environmental conditions, complex analytes, or biological processes. They have several advantages over traditional analytical methods and can be used as a feasible alternative to complex, offline instruments that are required in conventional assays [1]. Moreover, the toolbox of molecular biology techniques offers a plethora of possibilities for the design and adaptation of specialized biosensors with enormous potential.

**Figure 1:**
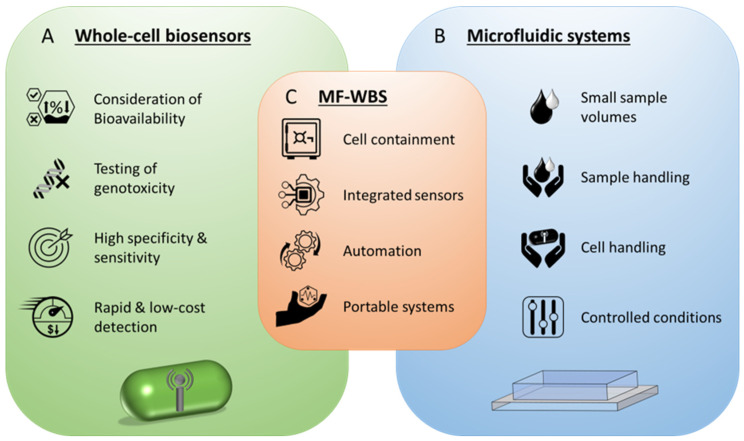
Overview of characteristic advantages and chances resulting from (**A**) whole cell biosensors and (**B**) microfluidic systems. (**C**) Chances of microfluidic whole-cell biosensor systems (MF-WBS) resulting from a synergistic combination of both techniques.

Whole-cell biosensors, also named microbial biosensors or cell-based biosensors, are a special subclass of biosensors, which use whole-cells as molecular recognition elements and for generating a quantifiable signal as output [2]. They can be of prokaryotic or eukaryotic origin and combine the biological receptor and the transducer in one component [1]. An amperometric, cell-based biosensor for ethanol determination is believed to be the first whole-cell biosensor [3]. It is based on the bacterium *Acetobacter xylinum* and was reported in 1975 [4]. Since then, many different whole-cell sensors for a wide range of analytes and environmental conditions have been developed [2,5]. Their various advantages were already discussed some decades ago [3,6,7,8] and are still relevant today [5,9] (Figure 1A). Some important advantages are, among others, the consideration of bioavailability of the target compound, reporting the bioavailable fraction in complex sample matrices, testing of genotoxicity, the ability to genetically engineer the biosensors to either highly specific compounds or for global sensing of environmental conditions (e.g., overall toxicity and oxidative stress), the avoidance of lengthy and expensive enzyme purification, enzyme preservation and protection, homeostatic mechanisms to adjust to environmental changes during measurement, rapid growth rates and low costs, and usage as multipurpose catalyst when multiple enzymes are required in the process [8,10,11,12,13].

Multiple approaches for the integration of such biosensors into suitable measurement setups were made to create functional and applicable analysis systems [14,15,16]. The increased emergence of microfluidic systems in the last decade has shifted the focus to miniaturization and the development of handy measuring devices. According to D’Souza, multidisciplinary approaches are required to reach the full potential of whole-cell biosensors in applications [17]. In this context, microfluidics is emerging as a promising tool, offering crucial advantages for measurement applications (Figure 1B). The advantages and possibilities arising from the characteristics of microfluidic systems are already successfully harnessed in novel system where it is used for cell handling (storing, sorting, immobilizing, capturing) and sample handling (storage, mixing, flow control) to develop rapid and sensitive detection applications with reduced reagent consumption and better control of detection conditions [18,19,20].

The symbiosis of whole-cell biosensors and microfluidic systems into microfluidic whole-cell biosensor systems (MF-WBS) offer some interesting chances for future sensorics, resulting from their inherent advantageous characteristics (Figure 1C). The integration in microfluidic setups enables safe containment and cultivation of the sensor cells overcoming the need of specialized immobilization techniques [5]. Moreover, microfluidic systems allow for integration of electrotechnical microsensors, thus offering a feasible platform for micro total analysis systems (µTAS) and automated sample handling and measurements. Furthermore, the miniaturization of the analytical devices facilitates the development of portable systems for point-of-care testing and on-field analysis [2,13,21]. Novel, innovative systems that harness the advantages of this symbiosis could push the concept of whole-cell biosensors into new dimensions. Different proof-of-concept systems have been developed throughout the years [22,23,24], nevertheless the technology readiness level of many systems is still rather low and further development faces many bottlenecks. Several challenges have to be overcome in order to design, manufacture and commission such systems.

In this study we investigate the challenges MF-WBS are currently facing during and after the development process, device implementation, and application. We assess the challenges based on their feasibility and rank them accordingly. Additionally, we explore potential future applications that arise from the opportunities these systems offer.

## 2. Challenges

**Figure 2:**
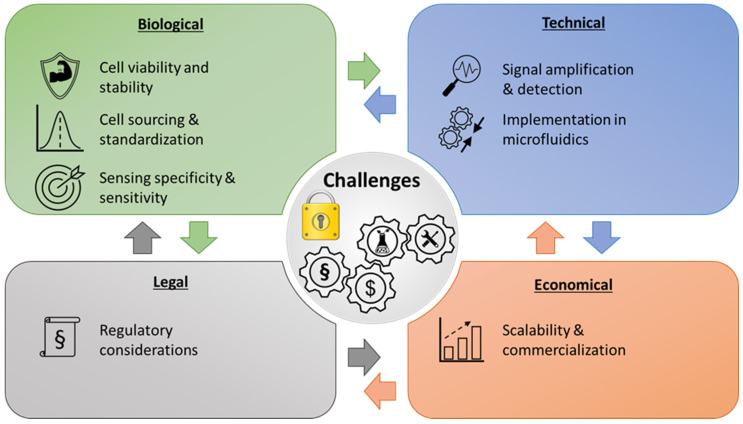
Overview of challenges for the conceptualization and establishment of microfluidic whole-cell biosensor setups as well as future relevant factors for further development and applicability in research and industry.

The reported MF-WBS demonstrate huge potential in monitoring, screening and point-of-care applications and can be used as examples for the relevance that small integrated whole-cell biosensors might have in future. However, there are many challenges that must be overcome for systematic and commercial usage. An overview of the main challenges as well as facilitating developments for future applicability are presented in Figure 2.

### 2.1. Cell Viability and Stability

Maintaining the viability and stability of living cells within microfluidic devices is crucial for accurate and reliable biosensing [14] and controlling the physiological activity can be a difficult task [25]. Cells may experience stress due to the microenvironment, such as shear stress, confinement, or exposure to different chemical gradients. Maintaining cell viability over extended periods, especially for long-term monitoring, is a challenge that requires careful optimization of culture conditions, nutrient supply, and waste removal. This also applies to storage possibilities and conditions of used sensor cells which is a crucial factor regarding viability, stability, and activity of organisms for field applicable point-of-care devices. The instability of the cells and their short shelf-life have been a general limitation for whole-cell biosensors [26]. The need for suitable preservation conditions was recognized some time ago and efforts were made to optimize these conditions, including varying culture conditions, storage protection media, sclerotization through desiccation, and lyophilization [27,28,29]. Other possibilities to maintain cell viability could be the integration of miniaturized incubators [14] or the usage of less demanding cell lines [30]. Bjerketorp et al. discussed different preservation (e.g., freeze- and vacuum-drying) and handling strategies, such as continuous cultivation and encapsulation, to maintain biosensor viability and activity, concluding that formulation and handling of sensor bacteria has to be considered carefully [31]. Even when optimal environmental conditions are selected to maintain cell viability, these conditions may impede the efficient and successful use of sensor cells due to differing requirements for analyte detection [32].

### 2.2. Cell Sourcing and Standardization

Cell sourcing and standardization of cell handling is closely related to cell viability and stability. Variability in cell lines or primary cells can affect the reproducibility and comparability of results. Reliable measurements with whole-cell biosensors therefore necessitate robust procedures for reproducible and standardized supply of cells for biosensing purposes. Ensuring consistent and well-characterized cell sources is essential for generating reliable data and facilitating meaningful comparisons between studies. Sometimes, certain cell types are required which can be difficult to obtain due to inconvenient processes or ethical concerns [33]. Differences due to different needs and requirements for cells in industry and research must be considered and possibly aligned. Important factors here are the storage and the initial state of the cells, as these influence various metabolic and genetic processes in cells and can therefore be responsible for unreliable analytical detection. Joshi et al. used a spore-based *Bacillus subtilis* biosensor for improved conservation possibilities that does not require freeze drying or refrigeration for storage. However, this had a negative impact on the quality of the biosensor in terms of sensitivity compared to their alternative *Escherichia coli* strain [34]. Furthermore, drift of bacterial metabolism, biofilm formation or contaminations could influence the measurements and thus the functionality of the system [35]. Reducing variability arising from cell sourcing and cultivation remains a major limitation and a critical challenge that must be addressed. Therefore, standardized calibration methods and protocols are required to mitigate this issue.

### 2.3. Sensing Specificity and Sensitivity

Achieving high specificity and sensitivity in microfluidic whole-cell biosensors can be challenging due to the complex nature of biological samples and potential interference from background signals. Furthermore, microorganisms are complex systems that might exhibit low selectivity in separate analyses, which can result in a composite signal across various toxic substances [36]. Ensuring that the biosensor system can selectively detect the target analyte amidst the presence of other molecules or contaminants requires careful consideration of the molecular recognition elements and optimization of the assay conditions. The sensitivity of some biosensors can be greatly impacted and drastically decreased by the chosen immobilization method [36,37] and is therefore a crucial element that must be taken into account. However, the severeness of the effect can differ between organisms and chosen immobilization materials [38]. Regarding microalgal-based biosensors, cell entrapment is considered to be the most critical element [24]. Furthermore, the small volumes and miniaturized devices require sensors and setups to be extraordinarily sensitive and precise in order to ensure reliable and reproducible analyte detection. However, some applications do not require a high specificity of the biosensor, but rather a broad analytical spectrum for the detection of substances in toxicity or drug screenings. In these cases, only biosensor sensitivity plays an important role that has to be regarded.

The biosensor sensitivity, however, can be influenced by the design of the microfluidic system, particularly the flow rates and structure geometry in flow-through systems can affect the sensing kinetics [39]. Continuous perfusion can accelerate analyte delivery and reduce diffusion limitations, possibly leading to faster response times. Additionally, it can stabilize the local analyte concentration by preventing depletion of the analyte and reducing accumulation of byproducts, which can improve signal stability. However, flow can also dilute or wash out analytes, metabolites, and signaling molecules, potentially lowering signal amplitude and thereby negatively impacting the signal-to-noise ratio, leading to reduced sensing specificity and sensitivity. Additionally, cellular behavior varies in a concentration-dependent manner [40]. The mentioned effects are system- and readout-dependent and do not universally apply. They must be carefully evaluated on a case-by-case basis for the respective application. Another essential factor for the sensitivity of the system is the signal transduction from cells to electronic components, which is influenced by the chosen substrate and the proximity of the cell to said substrate [41]. Attaining high sensitivity and specificity in microfluidic sensors continues to be a critical challenge [42].

### 2.4. Signal Amplification and Detection

Amplifying the cellular response to the target analyte and effectively detecting the resulting signal is another challenge. Depending on the target analyte, signal amplification strategies may be necessary to enhance sensitivity. Furthermore, integrating suitable detection methods, such as fluorescence imaging, electrochemical sensing, or mass spectrometry, with microfluidic platforms can be technically demanding and require careful optimization to achieve robust and reliable detection. Miniaturization processes further complicate the integration of technical sensors and reliable measurements at the detection limit. However, the performance of novel biosensors might rapidly be improved using statistical modeling approaches to increase sensitivity, signal output and sensing range of whole-cell biosensors [43]. Furthermore, the slow response time and therefore the slow analyte detection of some whole-cell biosensors can be a limiting factor [8]. Although this challenge may be tackled by permeabilization of cells with physical, chemical, or enzymatic methods [8], differences in chemical properties of water could affect the performance of whole-cell biosensors [44]. Moreover, these methods are unsuitable when maintaining cell viability and ensuring long-term operability are important. Sometimes cell immobilization is required to maintain cell viability for longer time periods, to ensure safe cell containment or to improve the performance of the biosensor. However the chosen immobilization technique can negatively impact the limit of detection [26]. The environmental conditions for cells during assay preparation or measurements are a relevant factor for signal detection and should be carefully considered.

### 2.5. Implementation in Microfluidics

The right implementation of whole-cell biosensors and technical microsensors in microfluidic devices plays a crucial role and is fundamental to the applicability of those setups in every respect as all challenges mentioned above have to be addressed and taken into account. The design and setup must ensure both functionality and safe entrapment of the whole-cell sensors while at the same time ensuring a physiological environment for extended periods that enables a rapid response to analytes and some form of signal transduction [45]. Especially the entrapment of cells can be a challenging part regarding the right design and material choices. Depending on the microorganism, common flow chambers that are used for adherent cells might not be applicable for other cells, i.e., cells with weak or no surface adhesion [46]. Devices made from borosilicate or silicon substrates require special treatment to ensure viable conditions for adherent cells [47]. However, material formulation and surface treatment can differ for each cell line and has to be optimized individually [48]. Cell damage and death is another challenge that can be caused by various reasons resulting from the microfluidic devices, such as mechanical stress due to the small scale nature, high shear stress, and cell interference induced by integrated controllers [47], thus impacting cell viability and signal detection. Further possible limitations are cell aggregation, leakage of devices, inefficient single-cell encapsulation as well as channel clogging and biofouling [42,47,49]. Another challenge is the miniaturization of the setups with simultaneous integration of various technical sensors under the same testing conditions as those specified in conventional scale assays, the complexity in manufacturing and handling, as well as the independence from laboratory connection and highly skilled personnel. Furthermore, choosing the right material is essential because it can drastically alter assay conditions and accuracy through interplay between plastics, adhesives, and other materials [50], e.g., absorption of small hydrophobic molecules in PDMS-based devices [51,52], or insufficient biocompatibility, e.g., possible effects of irritants used in the PDMS blends on cells [48] or leaching of uncured oligomers [51]. Alternative materials used for microfluidic devices, such as thermoplastics, also have drawbacks. Their fabrication can be more complex and costly in research settings [53], they may exhibit autofluorescence [54], bonding can be challenging [55], and achievable designs may be limited in terms of complexity and geometric accuracy [56]. However, if the balancing act succeeds and the implementation is successful, a huge potential can be exploited for broad applicability inside and outside the laboratory. This includes the implementation of easy and automated sample loading, handling, detection, and evaluation in a finished single device that can be manufactured and commercialized. Therefore, microfluidics plays an important role in developing said devices as it is an integral part for functionality regarding the design and development of small sample volume analyte detection in point-of-use applications.

### 2.6. Regulatory Considerations

As MF-WBS find applications in areas such as healthcare diagnostics or food safety, regulatory challenges need to be addressed. Compliance with regulatory requirements for safety, performance, and reliability is crucial to ensure the acceptance and adoption of these systems in clinical or commercial settings. The wide variety of designs and fabrication methods complicates standardization and regulatory approval due to the difficult establishment of quality control measures [42] and lacking standards and guidelines within the microfluidics community [57]. In particular, legal framework conditions for the storage, handling, and use of genetically modified organisms (GMO) represent a major challenge in the use and application of such systems, especially outside of regulated laboratory facilities. However, this not only presents challenges, but also a multitude of possibilities and opportunities due to the unique characteristics of microfluidic setups that could enable safe storage, handling, and usage of GMOs without releasing organisms. At the same time, the legal framework is also being revised and adapted to requirements and first genetically engineered biosensors are already approved for field use in Canada (NSN No. 19393) and the USA (MCAN No. J-18–0041) [16].

### 2.7. Scalability and Commercialization

If the full potential of microfluidic whole-cell sensor setups is to be exploited, miniaturization could lead to applications that are used outside the laboratory at the point-of-care. However, the transition of MF-WBS from research laboratories to practical applications requires much more than proof of concept. Despite the advantage of microfluidic solutions over many technologies for diagnostics, the generated revenue or profit is considerably low, or broad commercial markets do not exist [53], making it difficult to establish systems outside research purposes. Directing MF-WBS from laboratory environments towards commercial large-scale production for real-life industrial applications is one of the major challenges, as the systems need to be portable, durable, user-friendly, and consistent for testing at the point of care [58,59]. The scalability of production needs to be addressed to enable commercialization and widespread adoption. This includes the establishment of a suitable modern, precise, and profitable large scale manufacturing process as well as quality control, distribution possibilities, and storability. Especially the large-scale fabrication is difficult, regarding the complexity of fluid control at the given sizes [49] as well as the reproducibility and stability of whole-cell biosensors during long-term storage and transport [58]. Standardization of fabrication and cost-effectiveness are critical factors that need to be considered to ensure the practical implementation of these biosensor systems in daily use. Integration of whole-cell sensors and microelectronics into microfluidic devices often requires complex and elaborate manufacturing processes of said systems. The usage and distribution of frequently used genetically modified organisms are often associated with high costs and a large legal administrative burden regarding regulations and restrictions. This is of enormous importance when considering the scalability and possible commercialization approaches.

### 2.8. Challenge Overview

The list of potential challenges, bottlenecks, and limitations is extensive. Which hurdles arise during the development and commercialization of MF-WBS is not universal and depends on the specific application, requirements, target analyte, scaling considerations, and regulations. Table 1 gives an overview of the main challenges and resulting major limitations.

## 3. Challenge Impact and Potential Solutions

**Figure 3:**
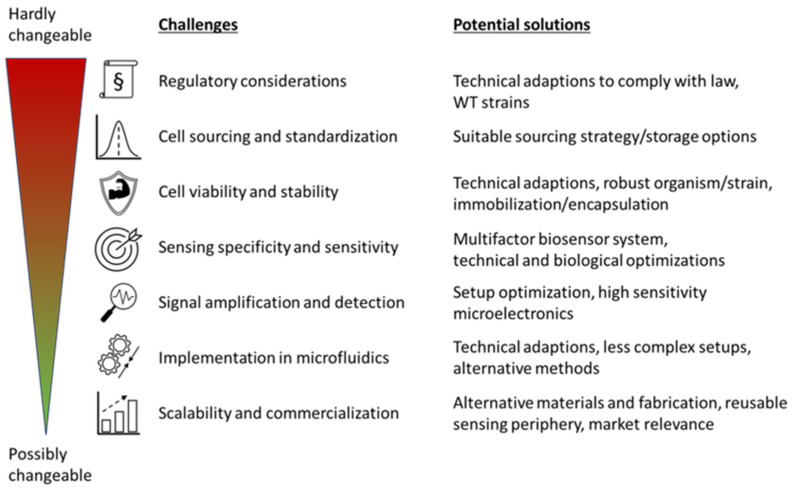
Ranking of the most critical challenges by their impact on applicability and potential solutions to overcome the bottlenecks and barriers. The challenges are ranked from hardly changeable (top, red) to possibly changeable (bottom, green).

In view of the many challenges to be overcome in the design and development of integrated microfluidic systems for measurement, screening and monitoring applications with whole-cell biosensors, the feasibility of various exclusion criteria must be considered and evaluated. Figure 3 shows the different challenges that could be possible bottlenecks and ranks them according to the possible effects they could have during conceptualization and development of novel setups. The ranking is based on a general assessment of each criterion and how likely it could lead to an unapplicable setup and is therefore case and application dependent when a specific setup is considered.

In general, a strong dependence of the relevance of the presented criteria on the respective situation and the preconditions given by the assay is to be anticipated. For example, the mandatory parameters of the systems to be miniaturized may cause individual criteria listed here to gain or lose significance, and possible solutions and countermeasures may also be null and void. Furthermore, the influence of the criteria shown on each other should also be mentioned. Adaptations and optimizations of one of the criteria can, for example, have an influence on the required prerequisites of another criterion or reduce the optimization possibilities by specifying a method.

Regulatory requirements and legal provisions are the be-all and end-all of any technology and method that is to be used. The possibility of using the conceptualized system stands and falls with them, as legal requirements, particularly the use of GMOs, are strictly regulated and new approvals and changes to the law are lengthy, expensive, and difficult to implement [60]. However, the field use and release of genetically modified organisms have been approved in the past [16,61], demonstrating the feasibility of establishing authorized systems. Furthermore, to decrease the regulatory burden suitable biocontainment strategies could be developed to ensure the safe on-site utilization of biosensors by preventing possible leakage into the environment, such as closed and self-sealing analysis systems [16] or cell encapsulation [62]. Another feasible strategy to address regulatory constraints is to design MF-WBS in a way that they do not fall under specific legal frameworks, thereby avoiding certain regulatory requirements altogether. In particular, restrictions associated with GMOs can often be circumvented by using wild-type (WT) strains. This approach has been demonstrated, for example, in MF-WBS for water toxicity analysis [24] and evaluation of pollutant toxicity [22]. Similar concepts can also apply to systems intended for medical or healthcare-related applications [63]. In this context, applications that are limited to, or inherently require, controlled laboratory environments are typically less critical from a regulatory perspective because compliance with biosafety and containment requirements for GMOs can be readily ensured under standard laboratory conditions (i.e., without environmental release). However, the use of WT organisms can be challenging because genetic modifications are often required to implement convenient signal readouts, such as fluorescence, bioluminescence, or colorimetric reporters. Therefore, adapting the readout strategy may be necessary to enable the use of WT strains. Several alternatives have been explored in the past, including the monitoring of growth-related parameters [14], cell motion/motility [64], or employing electrochemical readouts [28].

Cell sourcing and standardization as well as viability and stability are very crucial for successful implementation of MF-WBS. In contrast to legal regulations, they are more manageable with scientific research and resources. Changing the sensing mechanisms or used cell line can often be an adequate way to overcome complications associated with these criteria. However, sometimes certain organisms can be crucial for the given the assay conditions or are required to overcome regulatory specifications. Maintaining cell viability could be achieved using feasible electronics and microfluidic systems in combination with fluid control and autonomous feeding [33]. Other approaches pursue possibilities like freeze-drying [65,66], agar-immobilization [67], encapsulation [62], sclerotization (transformation process of plasmodial cells into a dormant form of the vegetative phase) [28], or spore formation [68,69] to increase shelf-life or maintain a high cell viability over extended time periods. Immobilization techniques can be of special importance as they can enhance the reproducibility, storage stability, and signal correlation to analyte concentration [26]. Regardless of which methodology is ultimately most suitable for a given application and therefore adopted, well-developed calibration methods and protocols are required to sufficiently standardize assays and measurements performed with whole-cell biosensors.

Sensing specificity and sensitivity can also be a challenge that can be tackled using suitable sensor organisms or refining the underlying biotechnological sensing principle. These exclusion criteria interact with the following criteria of signal amplification and detection. A lower sensing sensitivity could possibly be overcome with specialized equipment and technical sensors on the high-end scale. Several strategies to improve the sensitivity and robustness of MF-WBS have been proposed and, in part, implemented. For example, a three-leaf biosensor system was developed that integrates two independent biorecognition elements targeting the same analyte and includes an additional toxicity control within a single platform [70]. This configuration increased robustness and detection accuracy in complex samples without adding substantial workflow complexity. Moreover, the design enabled an early “rapid response” signal from one sensing element while the other readout was still developing, illustrating how multi-sensor integration can support both fast and reliable measurements in a single device. Another approach is to use different organisms or strains to increase biosensor sensitivity. Comparative studies using different microalgae in biosensor setups revealed pronounced differences in sensitivity between species [24]. It has also been suggested that high sensor-cell densities, particularly when combined with low pollutant concentrations, may reduce the apparent sensitivity of the readout. Consequently, optimizing cell density and key technical parameters should be considered early during MF-WBS development to maximize readout sensitivity. Another biological approach to improve the sensitivity of the readout could be to increase the sensor output by enhancing the reporter production via amplification circuits [62]. Aside from biological adjustments, technical parameters should also be carefully considered. Using highly sensitive microelectronic readout components is advantageous, and ongoing developments may further improve their performance. However, not only the sensing hardware but also the design, geometry, and implementation of the microfluidic setup can substantially influence selectivity and sensitivity [39]. Different approaches regarding technical sensors, biological modifications and assay optimizations could be exploited even at the same time to increase the sensing specificity and sensitivity.

The implementation of whole-cell biosensors in microfluidic setups remains a technical challenge. However, there has been a rising number of microfluidic systems with diverse applications in the past years. Technical advances facilitate manufacturing and open new possibilities to integrate biological and technical sensors. Despite the many possibilities and techniques that are already available, a remaining challenge is to find suitable combinations of biosensors, microfluidic setups and integrated microelectronics that allow for large scale production and applicability. Therefore, the conceptualization, design, and manufacturing of MF-WBS strongly interacts with the challenge of scalability and commercialization. Conventional manufacturing techniques, such as soft-lithography in combination with polymers, can be expensive, complicated, time consuming, and require special facilities as well as skilled personnel [24,71]. Thus, it may not be suitable for the upscaling to industrial levels and large-scale production [24]. Furthermore, materials like PDMS cannot be manufactured in large scale using high-throughput methods, such as injection molding, rolling, and embossing [53,72]. While the fabrication and use of PDMS-based microfluidic devices offer many advantages, making PDMS a widely used material for prototyping and academic research, the numerous potential biological and manufacturing limitations associated with PDMS can render it unsuitable for certain applications. Alternative materials for microfluidic devices have been explored, including polystyrene (PS) [73], cyclic olefin copolymer (COC) [73], polymethyl methacrylate (PMMA) [56,74], and polycarbonate (PC) [74] and demonstrated their feasibility for low-cost and high throughput production. However, this also means that the suitability of alternative materials must be evaluated on a case-by-case basis for each application. It is important to consider downstream product-development steps, such as manufacturability and scale-up, early on and to incorporate these constraints from the start [75].The excessive need for complex and expensive periphery to operate the microfluidics systems often led to failed in situ implementation of many of the existing microfluidic devices [24]. Alternative methods, such as xurography, could be used as easier and less expensive technique and moreover, even increase the functionality and applicability of such systems compared to conventional methods through a wide range of available materials [71]. Systems that are fully integrated or characterized by simplicity could be used by untrained personnel, which could further expand their applicability [76]. The progress made in the microfluidics sector over the past few years [42,47,59] as well as current developments will expand the possibilities and continue to ease the creation of novel systems.

The commercialization of MF-WBS remains a major challenge [58,59]. It often leads to a feedback loop regarding development and optimization steps that first has to be covered during the choice, design, and implementation of the sensors in suitable microfluidic systems as discussed above. Furthermore, commercial realization is always a tradeoff between the relevance or usefulness of the developed systems and the production costs that are influenced by the required materials and complexity of the system. In this context, manufacturing costs often represent the most significant barrier to the commercialization of these systems [72]. However, smart and simple designs of microfluidic systems, e.g., centrifugal disc systems [77,78], paper-based devices [79], as well as advances in microsensor technology [80] or the replacement of integrated electronic systems with easy available and accessible smartphones for signal readout [13], can reduce the cost and complexity of point-of-care tests. Thus paving the way for the production of simple, portable, and low-cost systems that can be operated by untrained users [13,77,78,80] and enabling fast and precise assays with a high degree of automation as well [81]. Furthermore, commercially available microchips and testing platforms might be used [82,83] in combination with simple or passive microfluidic principles to simplify and accelerate the commercialization of such systems. Economic efficiency can be improved by selecting alternative materials that reduce material costs and enable simpler fabrication and scalable manufacturing processes. This could be achieved by replacing materials commonly used in academic and laboratory settings, such as PDMS, with industrially preferred thermoplastics [75]. In addition, fabrication methods like xurography might be used for simple, rapid and low-cost fabrication of disposable microfluidic chips [84]. Easy to use systems can also increase the acceptance of the technology, which is often a hindrance for commercialization because it is an unfamiliar for many researchers and environmental practitioners [44]. Technological improvements could further propel the development of small wearable sensors that bear great commercial potential in medical diagnostics [12]. Therefore, scalability and commercialization are considered less relevant in the future compared to the other challenges, despite being a crucial challenge that could hinder real life applicability.

The presented challenge rating, however, navigates through a tangled web of issues, as each challenge strongly interacts with the others and varies depending on the specific task, process, situation, and requirements placed on the system that needs to be designed. Each individual approach can differ and may fail due to the requirements set by another challenge. Therefore, an assessment of the feasibility and applicability of a system is highly specific and should be well considered and evaluated in advance according to fixed parameters and requirements that cannot be replaced by alternatives.

## 4. Future Applications and Outlook

**Figure 4:**
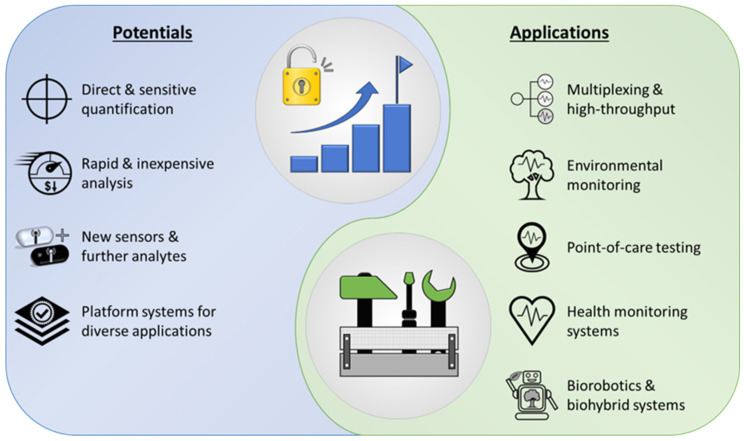
Overview of potentials that are unlocked using MF-WBS as well as relevant prospective and envisioned applications that could be covered with future systems.

If the existing challenges are successfully addressed, numerous future applications could emerge, resulting from the various potentials of whole-cell biosensors and microfluidic systems and the opportunities arising from their symbiosis (Figure 4).

Low sample volumes, automated sample handling, and preparation as well as the possibilities of parallelization and automation of workflows are of great interest for measurement applications in the laboratory and industrial sector. In addition, new application areas and measurement possibilities are emerging using whole-cell biosensors that are capable of directly and sensitively quantifying complex molecules, biological processes, or the effect of environmental influences on organisms. This way, time-consuming measurement processes, complex analytical procedures, and lengthy studies could be replaced by rapid and comparatively inexpensive analysis, provided that a scalable and cost-efficient manufacturing method is available for commercial production of such systems. Further developments in the field of whole-cell biosensors may open up new application areas in the future and provide a variety of novel sensors for the detection of diverse substances. Improvements of existing sensors could also help to increase their sensitivity to the analyzed substance or process. Moreover, existing MF-WBS and strategies could be used as a basis for the adaptation and combination of further biosensors. This platform principle enables the rapid further development of devices for new applications by integrating already characterized sensor strains into functioning microfluidic devices. Lu et al. even proposed adapting their UAV mounted biosensor device for enhancement of its functions by integrating other cell types and other optical and electrochemical readouts [14]. There is an abundancy of readily available biosensors that open a variety of possible detection, analysis, and monitoring possibilities [2,58]. A key challenge for the deployment of these sensors is to develop viable strategies to ensure compliance with local regulatory requirements and standards, and to broaden acceptance and facilitate regulatory approval. Some systems are not intended to be used as MF-WBS, but already fulfill some of the requirements and could easily be adapted. Nikkhoo et al. presented a rapid, accurate and reproducible assay for the detection of pathogenic bacteria [85]. This system could also be used vice versa for the detection of and the screening for novel bacteriocins to a certain organism.

The integration of new biosensors is of course accompanied by further developments in the field of microfluidic systems and their integration in existing biosensor systems to improve their applicability. There are some promising approaches with whole-cell biosensors that could profit from integration of microfluidics such as the algal conductometric biosensor that uses immobilized *Chlorella vulgaris* microalgae to test for alkaline phosphatase activity and toxic compounds [86]. Suitable biocompatible materials that also enable immobilization, along with efforts to maintain or improve sensing sensitivity, are therefore necessary. A feasible microfluidic setup could improve the applicability in terms of sample handling, detection automation and membrane regeneration for future detection and monitoring applications. New and sophisticated sensor technologies, such as impedance sensors, find their way into microfluidics and broaden the spectrum of possibilities [87]. Combined with manipulation methods such as negative dielectrophoresis (nDEP), this enables new approaches to detect and quantify cellular behavior at the microfluidic scale, provided that challenges in signal transduction, amplification, and detection are identified and mitigated.

Especially interesting are multiplexing approaches that enable high-throughput measurements, fast sample processing, and monitoring of diverse contaminants [88]. Functional toxicity screenings of numerous unknown analytes might be conducted on a single chip [89]. Multiplexed measurements on single-cell level for high-throughput screenings were already described about 20 years ago using a fiber-optic based microarray setup [90]. Integration of microfluidics and miniaturization of the described system could offer a feasible platform for optical biosensor applications with single-cell resolution.

Bioinspired environmental robots are an upcoming issue for remote environmental monitoring [91,92,93]. They could be used as platforms for the integration of microfluidic whole-cell biosensors where the biosensors are used for environmentally accurate sensing applications and microfluidics as crucial part for miniaturized low-power consumption setups and automated sample handling. Furthermore, biohybrid systems, such as artificial olfactory systems, offer a plethora of possible applications for environmental monitoring, public safety as well as applications in the medical, food and cosmetics industry where they could outperform traditional analytical methods in terms of cost efficiency, simplicity, accuracy and functionality [12,32,33]. To achieve this, miniaturizing and integrating the peripheral components required for microfluidic setups can be challenging and must be addressed accordingly. The integration of robotics and combination with whole-cell biosensors and microfluidics could revolutionize the automation of monitoring applications.

The possibilities are far from exhausted. Advances in sensor technology and microfluidic systems, as well as the integration of new and previously unused biosensors, hold enormous potential that has only been partially exploited to date. The ongoing miniaturization of systems as well as improved high-sensitivity and low-power technical sensors could serve as the basis for enabling widespread point-of-care testing in the future to the point of health monitoring and personalized medicine. At the same time, advancing digitization and networking of data can facilitate rapid analysis even in remote areas. In addition, image processing and machine learning are developing into powerful tools that will increase the influence of microfluidic systems in the future [59].

Today there are already many promising approaches for the integration of whole-cell biosensors into microfluidic system that make use of and demonstrate the enormous potential of this highly interdisciplinary field in environmental monitoring, medical testing, food safety, and many more. Given the current technological maturity and regulatory landscape, environmental monitoring is likely to be the first field in which MF-WBS are implemented on a larger scale. Environmental applications, such as detecting pollutants or heavy metals in water, benefit from comparatively lower regulatory barriers, simpler sample preparation than clinical diagnostics, and a strong need for portable and multiplexed detection platforms. In contrast, healthcare-oriented applications require stringent validation, robust biosafety concepts, and extensive regulatory approval, which will likely delay translation into practice. However, an urgent demand for point-of-care testing, driven by emerging diseases with substantial socio-economic impact (e.g., pandemics), could accelerate the development of these systems. In such contexts, MF-WBS may offer key advantages as novel diagnostic and monitoring approaches, potentially increasing acceptance and adoption and thereby enabling faster translation through prioritized evaluation and, where applicable, expedited regulatory pathways. Food safety represents another promising area due to the demand for rapid detection of pathogens and toxins. However, complex food matrices and requirements for industrial integration remain non-trivial hurdles. Overall, while key challenges, such as sensor stability, signal robustness, and biosafety, still need to be addressed, the underlying technological basis for these systems is already established. Once these limitations are mitigated, broader adoption in food safety and healthcare diagnostics is expected to follow. Nevertheless, most systems are still far away from real-world application and mainly find attention as proof-of-concept systems in academic research or usage in the near laboratory environment if already further developed. Often the storage, and complex handling of whole-cell biosensors in particular are exclusion criteria for the application of MF-WBS, which is why enzyme assays are preferred wherever possible. Further the required connection to laboratory equipment and systems such as FACS as well as the widespread usage of genetically modified organisms restricts the flexible and extensive usage caused by limited laboratory access, required specialist personnel, and socio-political restrictions in most countries. However, when those challenges are addressed and tackled in future, microfluidic whole-cell biosensors systems have the potential to revolutionize various industries by providing rapid, accurate, and cost-effective solutions for analyte detection and monitoring in diverse applications. Although the concept and idea as well as the possible advantages of the combination of whole-cell biosensors and miniaturized fluidic setups has been known for a while it is still an emerging field whose full potential has only been scratched so far. Progress in micro sensorics and electronics as well as simplified access to micromachining through high resolution 3D printing and CNC milling in recent years paves the way for the design, manufacturing, and testing of new functional setups. As researchers continue to optimize microfluidic as well as sensor concepts the capabilities and applications of MF-WBS will expand further. Therefore, modern techniques will ease the commercialization of point-of-care devices and could even make broadly available test kits for some applications in the near future.

## 5. Conclusions

Microfluidic whole-cell biosensor systems (MF-WBS) represent a cutting-edge approach to biosensing, using the unique properties of microfluidics and the inherent sensing capabilities of living cells. These systems offer extraordinary advantages such as enhanced sensitivity, specificity, throughput, real-time monitoring, and consideration of bioavailability. Often, however, the possible use is currently still restricted and limited by obstacles and technical challenges, so that a meaningful and profitable use in the future still requires further work on the simple implementation, changes in legal framework and reproducible biological standard procedures including the generation and usage of robust biosensor strains.

## Figures and Tables

**Table 1:** Overview of the presented challenges and respective major limitations and bottlenecks during development and application of MF-WBS.

Challenge	Major Limitations and Bottlenecks	Source
Cell viability and stability	Stress in microenvironment Stress through long-term monitoring Impact of storage and preservation methods on shelf-life Different requirements during analyte detection	[26,32]
Cell sourcing and standardization	Variability and heterogeneity of cells Conservation and storage conditions/requirements Biological characteristics (biofilm, cell cycle, etc.)	[34,35]
Sensing specificity and sensitivity	Immobilization method Biological and technical interference for complex samples Signal transduction Sensitivity of technical sensors	[36,37,41]
Signal amplification and detection	Technical sensor complexity Miniaturization Slow response time Cell immobilization and long-term operability	[8,26]
Implementation in microfluidics	Material (technical and biological constraints) Stress on cells caused by system Technical hurdles and bottlenecks (leaking, clogging, etc.) Integration of technical sensors Miniaturization of periphery Complexity of manufacturing and handling of systems	[42,47,49]
Regulatory considerations	Legal authorization and administration Regulations concerning storage and handling of GMOs Compliance with regulatory requirements	[16,42]
Scalability and commercialization	Low revenue, narrow commercial markets Development of user-friendly all-in-one devices Manufacturing methods (precision, throughput, cost) Storability and shelf-life	[49,53,58,59]

## Data Availability

No new data were created or analyzed in this study. Data sharing is not applicable to this article.

## Abbreviations

The following abbreviations are used in this manuscript:

µTAS	micro total analysis system
CNC	computerized numerical control
COC	cyclic olefin copolymer
FACS	fluorescence-activated cell sorting
GMO	genetically modified organism
MF-WBS	microfluidic whole-cell biosensor systems
nDEP	negative dielectrophoresis
PC	polycarbonate
PDMS	polydimethylsiloxane
PMMA	polymethyl methacrylate
PS	polystyrene
UAV	unmanned aerial vehicle
WT	wild type
